# Pan-cancer analyses of classical protein tyrosine phosphatases and phosphatase-targeted therapy in cancer

**DOI:** 10.3389/fimmu.2022.976996

**Published:** 2022-10-20

**Authors:** Tao Wang, Xinlei Ba, Xiaonan Zhang, Na Zhang, Guowen Wang, Bin Bai, Tong Li, Jiahui Zhao, Yanjiao Zhao, Yang Yu, Bing Wang

**Affiliations:** ^1^ College of Life and Health Sciences, Northeastern University, Shenyang, China; ^2^ Department of Pathophysiology, Bengbu Medical College, Bengbu, China; ^3^ Department of Thoracic surgery, The First Affiliated Hospital of Bengbu Medical College, Bengbu, China

**Keywords:** classical PTPs, GRNs, PTPscore, prognosis, drug-resistance

## Abstract

Protein tyrosine phosphatases function in dephosphorylating target proteins to regulate signaling pathways that control a broad spectrum of fundamental physiological and pathological processes. Detailed knowledge concerning the roles of classical PTPs in human cancer merits in-depth investigation. We comprehensively analyzed the regulatory mechanisms and clinical relevance of classical PTPs in more than 9000 tumor patients across 33 types of cancer. The independent datasets and functional experiments were employed to validate our findings. We exhibited the extensive dysregulation of classical PTPs and constructed the gene regulatory network in human cancer. Moreover, we characterized the correlation of classical PTPs with both drug-resistant and drug-sensitive responses to anti-cancer drugs. To evaluate the PTP activity in cancer prognosis, we generated a PTPscore based on the expression and hazard ratio of classical PTPs. Our study highlights the notable role of classical PTPs in cancer biology and provides novel intelligence to improve potential therapeutic strategies based on pTyr regulation.

## Introduction

Protein tyrosine phosphorylation is a dynamic and reversible post-translational modification regulated by protein tyrosine kinases (PTKs) and protein tyrosine phosphatases (PTPs). It is a fundamental mechanism for regulating innumerable biological processes (1, 2). Since several PTKs play an oncogenic role in activating growth factor-mediated cellular processes, PTPs are typically considered the negative regulators of these events and, accordingly, tumor suppressive. Nevertheless, accumulating evidence reveals that PTPs do not habitually counteract the activity of PTKs in catalyzing tyrosine phosphorylation but can also take prominent roles in the initiation and progression of signaling cascades that govern cell functions (3). Therefore, abnormal expression of encoding PTPs by genetic and epigenetic alterations can break the equilibrium of kinase-phosphatase activity, resulting in aberrant cell proliferation or cancer (4). Classical PTPs are a cysteine-based subclass of the PTP superfamily, including the transmembrane receptor-like and the intracellular nonreceptor proteins. Unlike some PTPs with more substrate specificity, including phosphothreonine and phosphoserine, classical PTPs strictly dephosphorylate phosphotyrosine (pTyr) in their substrates (5). Targeted methods effectively use PTK inhibitors that have previously been authorized for use in therapy; nevertheless, the therapeutic potential of regulating PTPs is still underexplored even though multiple PTPs have been identified as high-value targets (6, 7). Given the involvement of classical PTPs in human cancer, a more comprehensive understanding of them helps develop more efficient therapeutic interventions.

This study comprehensively characterized the molecular regulatory mechanisms and clinical relevance of classical PTPs in 33 types of cancer. We discovered that copy number variations (CNVs) are the predominant factor leading to abnormal expression of classical PTPs across cancer types. We further displayed the architecture and features of gene regulatory networks (GRN) by transcription factors (TFs) and microRNAs (miRNAs). In addition, we assessed the clinical prognosis for classical PTPs and constructed a rigorous model to evaluate the PTP activity in cancer. Finally, we explored the interaction network of classical PTPs with FDA-approved drugs and identified that classical PTPs are promising drug targets for improving therapeutic strategies.

## Materials and methods

### Human tissue samples collections

All human samples used in this study were collected from patients subjected to clinical surgery in the First affiliated hospital of Bengbu medical college. Before RNA isolation and protein extraction, samples were stored at -80 ℃. Collections of human samples were obtained and approved by the ethics committee of Bengbu medical college. Written informed consent was obtained from individual or guardian participants.

### Multi-omics data sources

The pan-cancer analyses were based on the Cancer Genome Atlas (TCGA) Research Network. We systematically analyzed 38 classical PTPs in 33 TCGA cancer projects. Every project indicates a particular cancer type. RNA-seq data for the transcriptional expression of 33 types of cancer were downloaded using the TCGAbiolinks R package (8). The somatic mutation data were acquired from the MC3 project of the TCGA PanCanAtla (9). The CNV data were downloaded from Broad GDAC Firehose in January 2016 (https://gdac.broadinstitute.org/). GISTIC2 was employed to define the significant gain or loss in genomic regions (10). The clinical data associated with TCGA patients were obtained from the published study (11) or downloaded using the TCGAbiolinks R package. The Chinese Glioma Genome Atlas (CGGA) (12), the Gene Expression Omnibus (GEO) database under the accession numbers GSE4290 (13), and the GlioVis dataset (14) were used to validate the results.

The omics data of cell lines, including gene expression profiles, somatic mutations, and CNVs, were acquired from the Broad Institute Cancer Cell Line Encyclopedia (CCLE) and the Genomics of Drug Sensitivity in Cancer databases (GDSC) (15, 16). The mutation and CNV frequency were defined as the proportion of cell lines with the variations in each cancer type (17, 18).

### Differentially expressed classical PTPs analysis

To avoid instability between tumor and normal samples in differential analyses caused by batch effect, we downloaded the gene expression profiles from the TCGA project, and the Genotype-Tissue Expression (GTEx) project that is re-computed by the UCSC Xena project depended on a defined pipeline. Thirty-eight differentially classical PTPs were acquired using the limma R package (19). The BH-adjusted p-value< 0.05 was regarded as the differentially expressed genes in each cancer type.

### Oncogenic pathway analysis across cancer types

Gene Set Variation Analysis (GSVA) was performed to compute the activity of 50 cancer hallmark-related pathways across 33 types of cancer. After that, the spearman correlation between the expression to classical PTPs and pathway activity was estimated. BH-adjusted p-valued< 0.05 and |Rs| > 0.3 were defined as significant.

### Construction of gene regulatory networks

To identify the interactions between miRNA and target genes (classical PTPs and TFs), we collected miRNAs from four target prediction databases: miRDB, miRTarBase, PITA, and TargetScan (20–23). The interaction pairs in at least two databases above were preserved for further research.

Next, we downloaded the promoter region sequence (from -1000 to +200 bp around the transcription start site) of target genes from the UCSC Table Brower depending on the human wgEncodeGencodeBasic V37 database. TF-binding sites were identified using the TFBSTools R package combined with JASPAR2020 datasets (24, 25). P-value< 0.00001 was retained.

Then, the feed-forward loops (FFLs) were extracted from the combination of miRNAs-genes and TFs-genes using MotifPredictor (publicly available at https://www.uth.edu/bioinfo/software.htm). The raw FFLs networks were further refined using TCGA expression profiles to improve the accuracy. The spearman correlation calculated TFs, miRNAs, and target genes for every FFLs in the raw network. For miRNAs-genes pairs, p-value< 0.05 and Rs< 0 was defined as significant. For TFs-genes pairs, p-value< 0.05 and Rs > 0 was defined as significant. The less significant FFLs networks were removed from raw networks to produce the final refined co-regulatory networks in each cancer type. In addition, the refined pan-cancer network was used to perform centrality measure and MCC analysis using the cytoHubba plugin (26), which was extracted modules using MCL that are both implemented in Cytoscape (version 3.7.2) (27).

Finally, we analyzed the correlation between classical PTPs and GO terms that contributed to nine cancer hallmarks as in previous studies (28, 29).

### Drug response analysis

The clinically actionable genes (CAGs) were retrieved from prior research as FDA-approved therapeutic drugs or biomarker targets (30). The therapeutic drugs and their known targets were adopted from the DrugBank database (31). CAGs with matching drugs as therapeutic targets were retained for further analysis.

We obtained the drug sensitivity area under the dose-response curve (AUC) and gene expression profiles for cancer cell lines from GDSC to examine drug sensitivity in cancer cell lines (32). We computed Spearman’s correlation between gene expression and the AUCs from GDSC and defined statistical significance as Spearman’s correlation coefficient |Rs| > 0.25 and FDR< 0.05. To evaluate drug response in TCGA patient samples, we retrieved the imputed tumor response to 138 anti-cancer drugs from prior research (33). We used Spearman’s correlation to determine the association between imputed drug response and mRNA, miRNA, protein, and DNA methylation of target genes. We defined statistical significance as |Rs| > 0.3 and FDR< 0.05.

### Clinical relevance and survival analysis

The survival analysis of classical PTPs was analyzed using the Kaplan-Meier method with the log-rank test by the survival R package. The cut-off point in each set was estimated using the survminer R package. P-value< 0.05 was defined as significant. Moreover, the Cox proportional hazard regression model was applied to estimate the hazard ratio (HR) for every classical PTP.

The log2 transformed expression profiles were first z-normalized for the classical PTPs with significant HR values across the analyzed samples (34). PTPscore was defined as the mean value of significant PTPs weighted by HR for each patient:


$$PTP\;score=\;\frac{1}{n}\sum_{i=1}^{n}e\;\;\times \;\;HR$$

where *n* is the number of analyzed samples, and e is the z-normalized expression data of involved PTPs with a significant HR.

To identify classical PTPs essential to the proliferation and survival of cancer cells, we applied CERES across 342 cancer cell lines (35). CERES is a computational method to estimate gene dependency levels from CRISPR-Cas9 essentiality screens while accounting for the copy-number-specific effect.

### Cell culture, transfection, and western blotting

BT-549, CAL-51, U-251 MG, and U-118 MG cell lines were cultured in Dulbecco’s modified Eagle’s medium (DMEM; HyClone, Thermo Fisher) supplemented with 10% fetal bovine serum (FBS; HyClone, Thermo Fisher) and 1% Penicillin/Streptomycin. U-87 cell line was cultured in Eagle’s Minimum Essential Medium (EMEM; HyClone, Thermo Fisher) supplemented with 20% fetal bovine serum and 1% Penicillin/Streptomycin. All cell lines were maintained at 37℃ in a humidified 5% CO_2_ chamber. Lipofectamine 3000 (Thermo Fisher) was applied to transiently transfection for plasmids and siRNAs following the manufacture`s instruction. After 48 h transfection, cells were lysed on the ice, and western blotting analysis was performed as previously described (36).

### Luciferase reporter assay

Cells were co-transfected with siRNA or plasmid and luciferase reporter plasmid containing the target promoter for 48 h. The luciferase activity was detected by the Luciferase Assay System (Promega, Madison, WI) and plate reader (BioTek, VT, USA).

### Cell cycle and apoptosis analysis

Cell cycle and apoptosis were analyzed using propidium iodide (1 mg/ml) and ribonuclease-A (10 g/ml) (PI/RNase; BD Biosciences), and Annexin V/PI assay by flow cytometry (BD Biosciences, Franklin Lakes, NJ, USA) respectively as we previously described (37).

### Cell proliferation assay

Cells were seeded in 96-well plates and evaluated with Cell Counting Kit-8 (CCK8; Bimake, Houston, TX, USA) according to the manufacturer`s instruction at the indicated time points. A plate reader (BioTek, VT, USA) was used at the endpoint to assess the results.

### Colony formation assay

Cells were seeded in six-well plates at a density of 1000 cells. After two weeks of growth, colonies were fixed with paraformaldehyde for 30 minutes and marked with 0.1% crystal violet solution for 15 min. Finally, an optical microscope was used to counter the number of colonies.

### Tumorigenesis in nude mice

Male mice (Four-week-old; BALB/c nude) were acquired from Charles River (Beijing, China) and fed in the house in the pathogen-free condition. All procedures were approved by the Institutional Committee on Animal Care of Northeastern University. U-257 cells stably expressing PTPN12 and empty vector were injected subcutaneously into nude mice’s right super lateral tissue (six mice per group, 2 x 10^6^ cells in serum-free DMEM). Mice were anatomized after two weeks. Western blot was used to detect the protein level of the target gene. The animal study was reviewed and approved by the Animal Care and Use Committee of Northeastern University.

### Statistical analysis

Statistical analysis and graphical visualization were performed in R, version 4.0.0 (https://cran.r-project.org/). The student’s t-test and the Wilcoxon rank-sum test were utilized to compare normally distributed variables and non-normally distributed variables. The p-values were two-sided and adjusted according to the Benjamini–Hochberg (BH) approach to control the false discovery rate (FDR). A BH-adjusted p-value< 0.05 was considered statistically significant unless otherwise indicated.

## Results

### Extensive dysexpression of classical PTPs in human cancer

**Figure 1:**
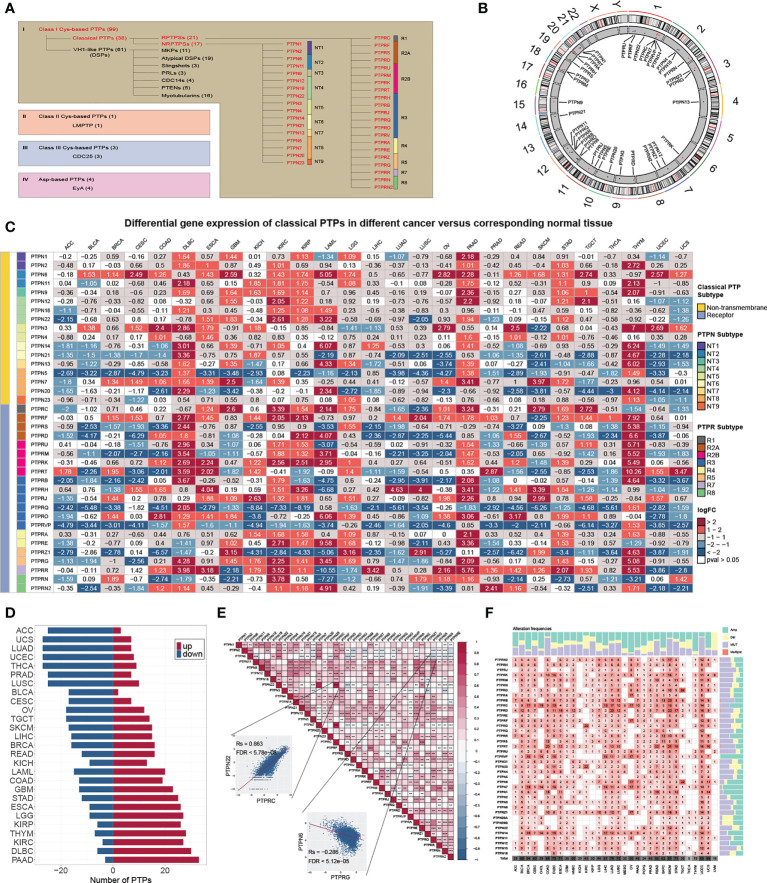
Genetic and expression landscape of classical PTPs in human cancer. **(A)** Classification of PTP superfamily. **(B)** Gene location on the chromosome. **(C)** Differential gene expression of classical PTPs across 27 types of cancer. **(D)** The proportion of significantly dysregulated classical PTPs for each tumor. **(E)** Correlation among the expression of classical PTPs in pan-cancer. **(F)** Detailed heatmap of alteration frequencies.

In this study, we reviewed the literature and explored the roles of classical PTPs in human cancer (**Figures 1A, B**). We first quantified the differential expression of classical PTPs in cancers by integrating the TCGA and GTEx data and found that the abnormal expression of classical PTPs was universal (**Figure 1C**). We further exhibited that some cancer types expressed a higher ratio of up-regulated PTPs (such as pancreatic adenocarcinoma (PAAD) and diffuse large b-cell lymphoma (DLBC)), and other cancers had more down-regulated PTPs (such as adrenocortical carcinoma (ACC) and uterine carcinosarcoma (UCS)) (**Figure 1D**). Since genes do not work in isolation, several PTPs may participate in an event simultaneously or not (2). We thus inspected the expression correlation among classical PTPs and discovered significant expression patterns across 33 cancer types (**Figure 1E**). For instance, PTPN22 was positively correlated with PTPRC (Rs = 0.863, FDR< 5.78 × 10^-8^), and PTPN6 was negatively correlated with PTPRG (Rs = -0.286, FDR< 5.12 × 10^-5^).

To characterize the genomic alterations of classical PTPs, we identified their somatic mutations and CNVs frequency across 33 cancers (**Figure 1F**). The general average mutation frequency of classical PTPs was low, ranging from 0.00 to 27.59% (90% classical PTPs< 4.38%) (**Figure S1A**). Several PTPs, like PTPRT, PTPRB, and PTPRD, demonstrated a high mutation rate in skin cutaneous melanoma (SKCM) and uterine corpus endometrial carcinoma (UCEC) compared with other cancers due to the higher global mutation burden in the two cancer types. These results showed that the somatic mutations were not the primary driver for the abnormal expression of classical PTPs. Moreover, we systematically explored the CNV alteration frequency for classical PTPs and observed that CNV alterations are widespread (**Figure S1B**), especially in ovarian serous cystadenocarcinoma (OV) and uterine carcinosarcoma (UCS) (**Figure S1C**). We also found that PTPN23 showed the most widespread CNV alterations across cancer types (**Figure S1D**). In contrast to PTPN23, the dysregulation of PTPRN was not significant with CNVs across cancer types (**Figure S1E**). Interestingly, the abnormal up-regulation of some classical PTPs was associated with CNV gain such as PTPRA in rectum adenocarcinoma (READ) (Rs = 0.81, FDR< 1.68 × 10^-32^) and PTPN2 in UCS (Rs = 0.74, FDR< 5.53 × 10^-9^), and some abnormal down-regulations were associated with CNV loss such as PTPN23 in UCS (Rs = 0.66, FDR< 8.23 × 10^-7^) and PTPRS in OV (Rs = 0.53, FDR< 5.78 × 10^-19^) (**Figure S1E**). These results suggested that CNV is the dominating event for dysregulation of classical PTPs in human cancer.

### Gene regulatory networks of classical PTPs

**Figure 2:**
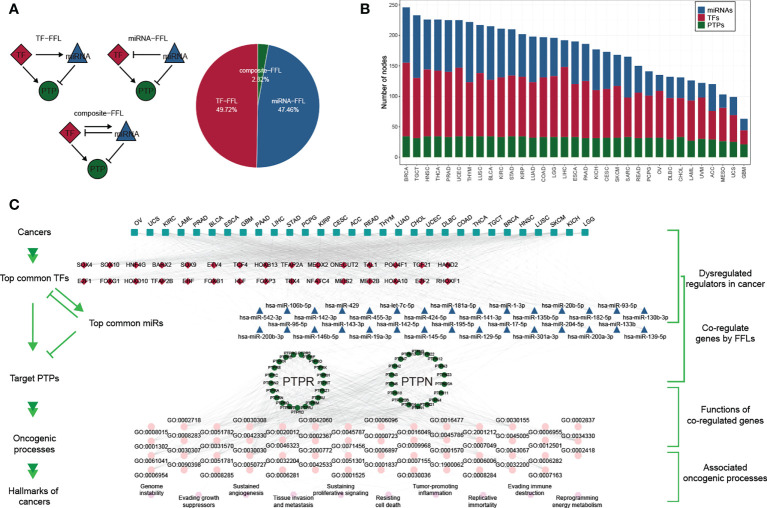
Gene regulatory network of classical PTPs across cancer types. **(A)** Three kinds of FFLs and matching proportions in pan-cancer. **(B)** Distributions of TFs, miRNAs, and PTPs in FFLs across 33 cancers. **(C)** A hierarchical model for cancer hallmarks and functional regulators.

To further explore the gene regulatory mechanisms of classical PTPs, we constructed the gene regulatory networks based on TFs and miRNAs. We divided the feed-forward loops (FFLs) into three categories referenced in the published study (38), including TF-FFLs (the TFs only directly regulate the miRNAs), miRNA-FFLs (the miRNAs only directly regulate the TFs), and composite-FFLs (the TFs and the miRNAs regulate each other) (**Figure 2A**). The distribution of the nodes in each FFLs was shown in **Figure 2B** across 33 types of cancer. Before constructing the hierarchical model, the representatives of classical PTPs targeted by TFs and miRNAs were characterized and validated, respectively. The network for TFs-PTPs pairs was illustrated (**Figures S2A, B**). 66 TFs were implicated as potential regulators of classical PTPs across cancers (**Figure S2C**). Some TFs are associated with multiple PTPs, such as several tumor metastasis regulators (ZEB1, SNAI3, and SMAD2). Since PTPN2 had the most potential TFs, we examined the correlation between the expression of PTPN2 and their potential TFs in 33 cancers (**Figure S2D**). Combined with chip-seq data acquired from Cistrome Data Browser (39) and molecular experiments, we proved that NRF1 is the accurate TF for PTPN2 in breast cancer cell lines (**Figures S2E–H**). Meanwhile, the features and validations of miRNAs-PTPs pairs were also identified (**Figures S3A–F**). In brief, these results strengthen the veracity of GRNs for classical PTPs.

To describe the regulatory mechanisms of GRNs in extenso, we constructed the co-regulatory network and integrated TCGA expression data to refine the FFL patterns (**Figure S4A**). The core subnetwork generated by the MCC algorithm contained 50 nodes (including five miRNAs, 23 TFs, and 22 PTPs) and 203 interactions (**Figure S4B**). We also got three modules using the MCL algorithm adjacent to network topology (**Figures S4C–E**). The detailed networks of top frequency classical PTPs were displayed (**Figures S4F–I**).

Despite the regulatory complexity and tumor diversity, several cancer hallmarks can promote the efficient development of human cancers. By calculating the correlation between GRNs and cancer hallmarks, we constructed a hierarchical model to interpret the contribution of classical PTPs to cancer progression (**Figure 2C**).

### Functional effects of classical PTPs

**Figure 3:**
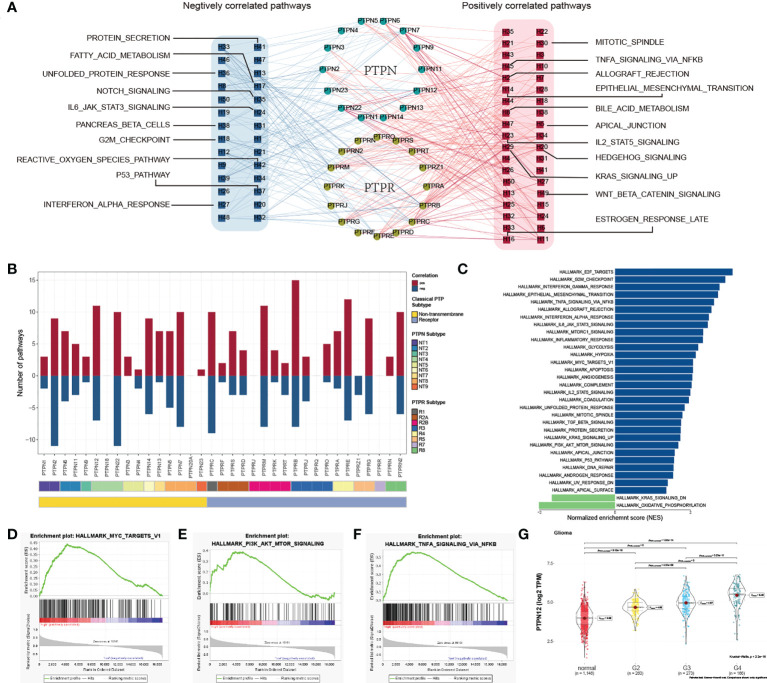
Functional analysis of classical PTPs in cancer-hallmark pathways. **(A)** Network of correlation between classical PTPs and cancer-hallmark pathways. **(B)** The number of pathways of involved PTPs. **(C)** Distribution of normalized enrichment scores for pathways using GSEA analyzing PTPN12 functions. **(D–F)** GSEA enrichment plot of the representative gene sets for PTPN12. **(G)** Relationship between the PTPN12 expression and clinical-grade in glioma.

To characterize the functions of classical PTPs in cancer progression, we performed the correlation analysis between the expression of classical PTPs and the activities of 50 cancer hallmark-related pathways. We identified that classical PTPs were associated with activating or suppressing multiple oncogenic pathways (|Rs| > 0.3, FDR< 0.05; **Figure 3A**). For example, the expression of PTPRB, PTPRE, and PTPN12 was associated with the activation of several oncogenic pathways. However, PTPRZ1 was exclusively enriched in the inhibited pathways, which followed the character of the suppressive gene (**Figure 3B**).

Taking PTPN12 as an example, due to the significant correlation with cancer progression (40), we validated the potential function in glioma using GSEA and observed that PTPN12 contributed to several oncogenic pathways (**Figure 3C**), including PI3K-AKT-mTOR signaling, and TNFα signaling (**Figures 3D–F**). Further analysis found that PTPN12 was up-regulated in human glioma from TCGA and other independent datasets (**Figure S5A**). In addition, the mRNA expression of PTPN12 displayed a strong association with the advanced grade and short survival time in glioma (**Figure 3G**; **Figure S5B**). To better address whether PTPN12 was correlated with glioma tumorigenesis, we applied clinical specimens and observed that PTPN12 was significantly overexpressed in glioma samples contrasted with the paired adjacent samples at the protein level (**Figure S6A**). The ectopic expression and knockdown experiments were performed to evaluate the effect of PTPN12 on cell growth. We found that the overexpression of PTPN12 notably enhanced the growth velocity in different glioma cell lines (**Figure S6B**), while PTPN12 silencing inhibited cell growth (**Figure S6C**). Consistent with the above results, PTPN12 promoted colony formation and tumor growth in nude mice (**Figures S6D–G**). Finally, we performed cell cycle and apoptosis analysis on PTPN12 in glioma cells. We found that PTPN12 could promote the proliferation and inhibit apoptosis of glioma cell lines (**Figures S6H, I**). Furthermore, Chen et al. got the results like us. They found that, unlike epithelial cell-derived carcinomas, PTPN12 in glioma, primarily derived from neural stem cells, suppressed migration/invasion but promoted growth and viability even though EGFR and HER2 are hyperphosphorylated (41). Taken together, these results suggest that PTPN12 is a potential oncogene in glioma and may be a promising target for glioma treatment.

### Integrative analysis of classical PTPs on drug response

**Figure 4:**
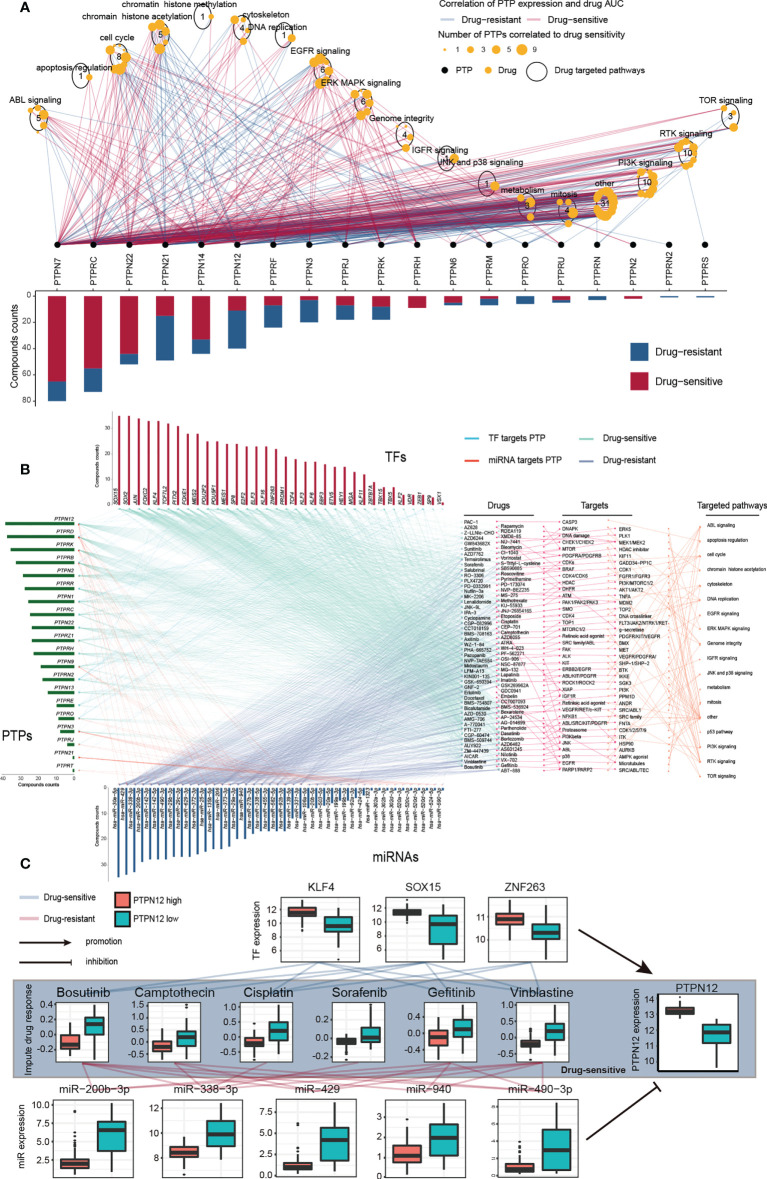
Significant correlation of classical PTPs to drug response in GDSC and imputed data from TCGA. **(A)** Spearman correlation between mRNA expression of classical PTPs and drug sensitivity across 1,074 cancer cell lines. **(B)** Sensitive interaction network of classical PTPs, PTP-targeted TFs, PTP-targeted miRNAs, and drug response in TGCT. **(C)** Representative drugs correlated to PTPN12 and corresponding TFs and miRNAs in TGCT.

To evaluate the potential responsiveness of classical PTPs to anti-cancer drugs, we explored Spearman’s correlation between the expression of classical PTPs and drug sensitivity for 252 anti-cancer drugs from the GDSC dataset across 1,074 cancer cell lines (32). The involved drugs target various biological processes, including DNA replication, apoptosis regulation, and EGFR signaling. We discovered 19 PTPs that significantly associated with the sensitivity of a total of 124 anti-cancer drugs (|Rs| >= 0.25, FDR< 0.05; **Figure 4A**; **Figure S7**). For instance, the mRNA expression of PTPN12 was up-regulated in ten types of cancer, and connected with drug sensitivity to 9 anti-cancer drugs (e.g., Trametinib, Rs = -0.38, FDR< 3.7 × 10^-20^) and linked to drug resistance to 33 anti-cancer drugs (e.g., THZ-2-102-1, Rs = 0.32, FDR< 7.1 × 10^-23^). Dysregulation of the EGFR signaling pathway is an established feature in multiple cancer types, and EGFR signaling can be regulated by classical PTPs (42). In our analysis, six EGFR signaling pathways drugs were significantly associated with 12 classical PTPs (**Figure S7A**). Our results across cancer cell lines showed extensive interactions between classical PTPs and drug response, highlighting the potential of combining anti-PTP drugs with other cancer therapies.

To further explore the impacts of classical PTPs on therapeutic response, we conducted an integrative analysis to assess the connections between the gene regulatory network of classical PTPs and response to anti-cancer therapies in TCGA patients. Taking testicular germ cell tumor (TGCT) as an example, we identified 20 PTPs that are highly associated with the sensitivity of 94 anti-cancer drugs and are targeted by 34 TFs and 42 miRNAs. The expression of these PTPs and paired TFs is negatively associated (drug-sensitive) with the response to anti-cancer drugs. In contrast, their paired miRNAs are positively associated (drug-resistant) with identical drugs (**Figure 4B**). The involved drugs target several pTyr regulation pathways, including EGFR and RTK signaling. For example, recent research identified the regulation of PTPN12 in EGFR signaling and RTK signaling (43, 44), and PTPN12 expression negatively associated (drug-sensitive) to the response to the EGFR signaling pathway inhibitor Gefitinib (EGFR, Rs = -0.41, FDR< 3.0 × 10^-7^) and RTK pathway inhibitor Axitinib (PDGFR, KIT, VEGFR, Rs = -0.63, FDR< 7.04 × 10^-18^), Lestaurtinib (FLT3, JAK2, NTRK1, RET, Rs = -0.59, FDR< 4.94 × 10^-15^), Sorafenib (PDGFRA, PDGFRB, KDR, KIT, FLT3, Rs = -0.46, FDR< 3.19 × 10^-9^) and PD-173074 (FGFR1, FGFR3, Rs = -0.45, FDR< 7.11 × 10^-9^) (**Figure 4C**). Meanwhile, three TFs target PTPN12 are also negatively associated with the corresponding drugs, but five miRNAs are positively correlated. For drug resistance of classical PTPs in TGCT, we also detected a similar gene regulatory network strongly associated with drug resistance to anti-cancer drugs (**Figures S8A, B**). Taken together, our findings implied that classical PTPs have a complex effect on drug response, the different drug responses to anti-cancer drugs might lead to opposite therapeutic outcomes.

### Application of classical PTPs in immunotherapy

**Figure 5:**
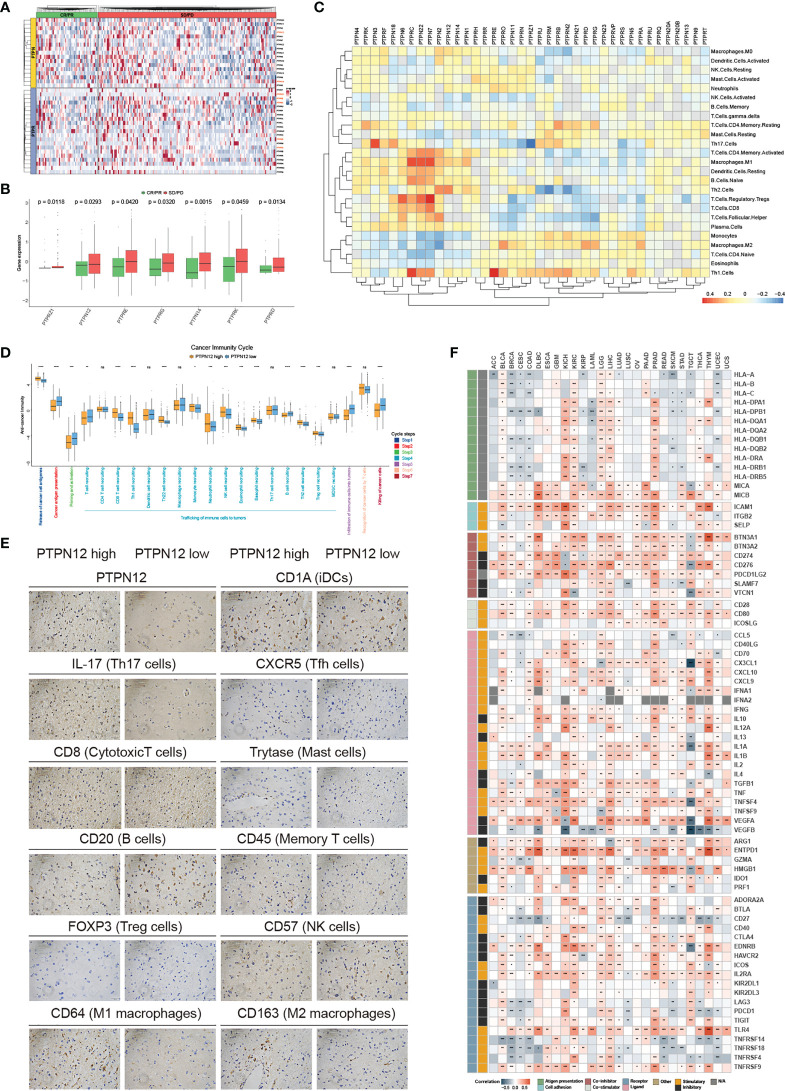
Immunological characteristics of classical PTPs in the TME. **(A)** Relative expression of classical PTPs in the responder (green) and the non-responder (red) subsets. **(B)** Significantly different PTPs between the responder and the non-responder subsets. **(C)** Correlation between classical PTPs and immune cell infiltration. **(D)** Deviations for the cancer immunity cycle between the high- and low-PTPN12 groups in glioma. **(E)** Representative IHC images of infiltrated immune cells in glioma. **(F)** Correlation between PTPN12 and 129 immunomodulators. *P < 0.05, **P < 0.01, ***P < 0.0001, ****P < 0.00001. ns indicates not significant.

Immunotherapy has become an efficient clinical approach for cancer treatment. We, therefore, estimated the roles of classical PTPs in anti-PD-L1 treatment (**Figure 5A**) and found that seven PTPs were significantly higher in the non-responsive tumor, including PTPN12 and PTPRE (**Figure 5B**). These results indicated that up-regulation of specific PTPs might donate the resistance to PD-1 blockades. Moreover, recent studies highlighted the role of PTPs in modulating the immune infiltrate, the relationship between the expression of classical PTPs and the immune cell infiltration was calculated (**Figure 5C**). To mechanically explore the functions of classical PTPs in shaping the TME, we evaluated the cancer immunity cycle process of PTPN12 in glioma (**Figure 5D**). We identified that PTPN12 was associated with the infiltration of several immune cells, such as Th17 cells, M1 macrophages, and cytotoxic T cells (**Figure 5E**). We also observed that PTPN12 was positively correlative with most immunomodulators across cancer types (**Figure 5F**). These observations provided several ways to understand classical PTPs on intervening immunotherapy and patient survival.

### Clinical implications of classical PTPs and PTPscore

The characteristics of classical PTPs may provide important insight into the development of clinical transformation. We observed that classical PTPs showed significant associations with patient survival and could be an excellent prognostic biomarker in various cancers (**Figure S9A**). Some classical PTPs exhibited carcinogenic features (**Figure S9B**), such as PTPN12 in PAAD (**Figure S9C**) and PTPN6 in acute myeloid leukemia (LAML). The chi-squared test revealed that patients with high PTPN12 expression had worse clinical staging and short lifetime, indicating that highly malignant PAAD is related to high expressed PTPN12. In contrast, several PTPs were identified as the suppressive genes in cancer, such as PTPRZ1 in BRCA (**Figure S9D**) and PTPRQ in lung adenocarcinoma (LUAD). High expression of these potential tumor suppressors may improve patient survival (45, 46). Further analysis found that the carcinogenic and CNV-driven PTPs showed more significant cell proliferation inhibition after knockout than other PTPs (**Figure S9E**).

**Figure 6:**
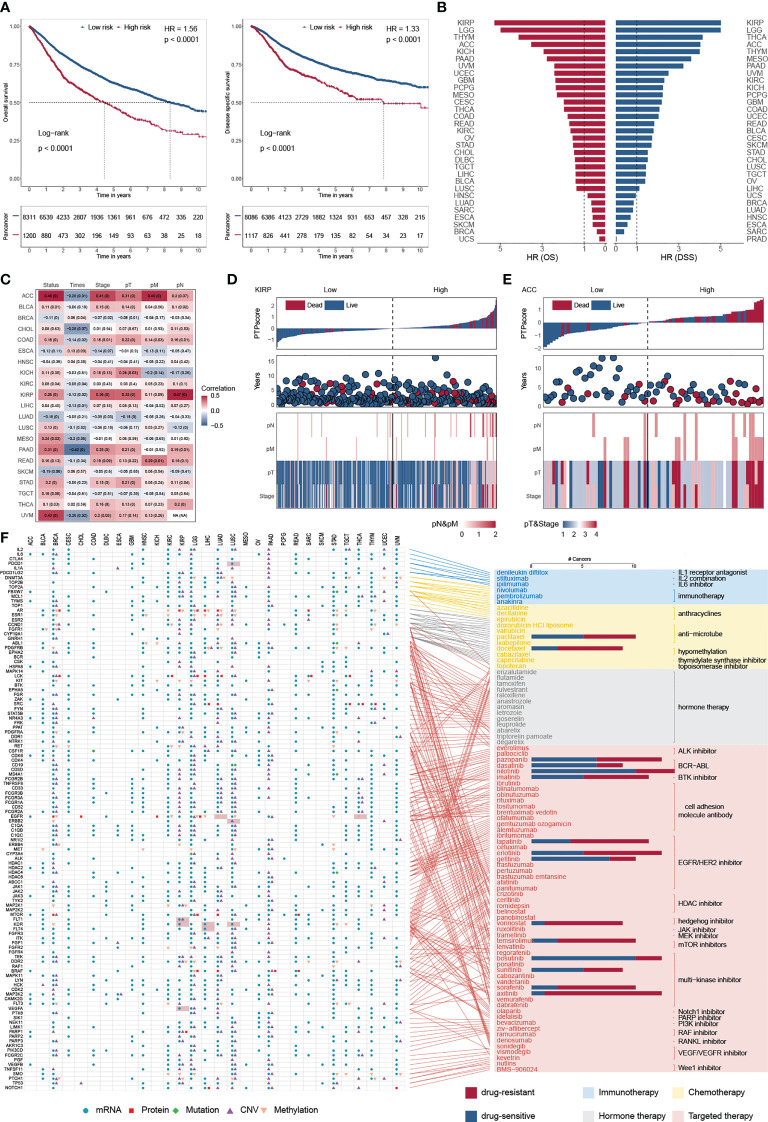
Predictive ability of PTPscore across cancers. **(A)** Kaplan–Meier plot showing the 10-year OS (left) and PFS (right) for PTPscore in pan-cancer. **(B)** The HR of OS and DSS for PTPscore in different cancer. **(C)** Correlation between PTPscore and AJCC stage. **(D, E)** PTPscore predicted values distribution with patient survival status (top), survival years (middle), and AJCC TNM staging status (bottom) for KIRP and ACC. **(F)** Association between FDA-approved drugs and their linked CAGs (right) and alterations of these genes at mRNA, protein, DNA methylation, mutation, and SCNA levels based on PTPscore across cancers (left). Different symbol shapes indicate various types of molecular signatures. Filled cells suggest that the gene is a therapeutic target of clinical practice in the corresponding cancer type. Bar plots in the right panel indicate the number of cancer types with a positive correlation (drug-sensitive, blue) and negative correlation (drug-resistant, red) between PTPscore and drug response (Spearman’s correlation).

Overall survival (OS) is widely regarded as the gold standard in cancer research, having the most straightforward and clinically meaningful endpoint definition: dead or alive. Furthermore, disease-specific survival (DSS) was superior to OS in patient outcomes (47). To explore the impact of PTP activity on cancer progression, a 10-year OS and DSS analyses were applied based on the PTPscore (**Figure 6A**). We observed a strong correlation between the high PTPscore and the poor 10-year OS and DSS. We further investigated the clinical relevance of PTPscore and identified that PTPscore exhibited an excellent predictive ability in multiple cancers (**Figure 6B**). The American Joint Committee on Cancer’s (AJCC) TNM staging method is universally acknowledged as a high predictor of therapy response and survival in human cancer. Thus, we calculated the correlation between PTPscore and pathological tumor TNM (pTNM) stage as the same as survival information (**Figure 6C**). Our analyses showed that a high PTPscore significantly correlated with the advanced stage and poor survival status, especially in kidney renal papillary cell carcinoma (KIRP) and ACC (**Figures 6D, E**). These findings suggested that the PTPscore is a good indication of prognosis and survival in multiple cancers.

To further delineate the clinically practical feasibility of PTPscore, we explored the multidimensional alterations of 118 CAGs targeted by 86 FDA-approved drugs between high-PTPscore and low-PTPscore patients across multiple cancers (30) (**Figure 6F**; **Figure S10A**). We identified PTPscore-associated features, ranging from one feature in cholangiocarcinoma (CHOL) to 189 in lower grade glioma (LGG) (**Figure S10B**). For instance, EGFR were biased in PTPscore-high subgroups with mRNA alteration in eight cnacers (e.g., ACC, logFC = 1.42, FDR< 1.64 × 10^-10^; LGG, logFC = 1.50, FDR< 3.13 × 10^-10^), protein alteration in three cancers (e.g., CHOL, logFC = -1.34, FDR = 0.016; LGG, logFC = 0.83, FDR< 3.13 × 10^-10^), CNV alteration in two cancers (PAAD, FDR< 1.15× 10^-10^;LGG, FDR< 4.45× 10^-12^) and methylation alteration in nine cancers (e.g., LUAD, logFC = 0.18, FDR = 0.004; liver hepatocellular carcinoma (LIHC), logFC = 0.48, FDR< 2.55× 10^-11^). Surprisingly, 98.3 percent (116/118) of CGAs were connected with at least one kind of PTPscore-associated molecular signature in at least one cancer type. Interestingly, PTPs has an effect on a number of immunotherapeutic targets. PDCD1 (PD-1) was shown to be strongly expressed in PTPscore-high LUSC samples (logFC = 0.58, FDR< 4.66 × 10^-11^), suggesting that PDCD1 inhibitors, such as nivolumab and pembrolizumab (48, 49), may be more effective in PTPscore-high tumors.

We next assessed the PTPscore on drug response using the imputed drug data from the TCGA samples. We observed that the response to sorafenib was positively associated (drug-resistant) to PTPscore in BRCA (Rs = 0.42, FDR< 3.94 × 10^-43^; **Figure S10C**), which is in line with the resistance research in breast cancer cell lines MCF7 and MDA-MB-231 and leads to the failure of the clinical trials in phase III (50). We further found that the response to Nilotinib was negatively associated (drug-sensitive) with PTPscore in UVM (Rs = -0.69, FDR< 6.13 × 10^-12^), which is in line with the Phase II multicenter trial in melanoma patients (51). These findings imply that our research is dependable and yields significant clinical insights. Patients with high-PTPsocre are resistant to multiple drugs, such as Vorinostat in THYM (Rs = 0.77, FDR< 3.14 × 10^-24^) and Axitinib in LIHC (Rs = 0.43, FDR< 1.04 × 10^-9^), indicating a possible therapeutic advantage of combining cancer therapy with phosphatase-targeted therapy for individuals with THYM or LIHC. Strikingly, some high-PTPsocre tumors may develop sensitivity to other drugs, including Gefitinib in uveal melanoma (UVM) (Rs= -0.82, FDR< 1.45 × 10^-19^) and AZD6244 in TGCT (Rs = -0.68, FDR< 9.06 × 10^-19^), which shows that phosphatase-targeted treatment may not be beneficial for patients with these malignancies.

## Discussion

Classical PTPs have been reported to play a role in tumorigenesis and be designed as a drug target for cancer therapy (52, 53). Learning in-depth about the molecular characterization and clinical relevance of classical PTPs is conducive to understanding cancer biology and improving clinical treatment. By exploring the TCGA multi-omics profiling data, we comprehensively and systematically illustrate the landscape of molecular regulatory mechanisms of 38 classical PTPs across more than 9000 patients from 33 types of cancer. Because protein tyrosine phosphorylation acts as both a tumor suppressor and a tumor promoter in different contexts, phosphatase function is unclear. However, given its significant involvement in cancer, it will be vital to understand its activity in various contexts to develop therapies for cancers with abnormal tyrosine phosphorylation. As a result, our results highlighted the notable role of classical PTPs in cancer biology and provided novel intelligence to improve potential therapeutic strategies based on pTyr regulation.

In this study, we observed the widespread abnormal expression of classical PTPs in human cancer and wondered what factors contribute to it. We examined the somatic mutations and found that classical PTPs displayed a low mutation rate in most cancers, probably attributed to the highly conservative PTP superfamily. However, PTPRT showed higher mutation frequency in cancer, which accorded with several studies (54, 55). Moreover, the enzymatic activity of classical PTP is mainly regulated by Cys and Asp in the catalytic domain (e.g. Cys229 and Dsp197 in PTPN18; Cys227 and Dsp195 in PTPN22) (56). Due to the highly conserved properties of classical PTP in cancer, the mutation frequency of these sites is very low, so the expression level is the main factor affecting the enzymatic activity. We further found that the high frequency of CNVs alterations is the main factor responsible for the dysregulation of classical PTPs. These results implied that CNVs alterations could change gene dosage and contribute to tumorigenesis or aberrant cell proliferation. For example, in this study, PTPRF, defined as the CNV-driven gene, was frequently amplified and up-regulated in multiple cancer. PTPRF was also associated with poor survival in ACC, LAML, LGG, and SKCM, which agreed with several studies (57, 58). Interestingly, some studies regarded PTPRF as the potential predictor for treatment with Erlotinib in lung cancer (59), indicating the clinical application of PTPRF in cancer therapy. In addition, we observed that PTPRF was co-amplified or co-deleted with PTPRK and PTPN3 in various cancers. PTPRK was reported to inhibit tumor progression by directly targeting STAT3 activation (60). PTPN3 could suppress cell growth and metastasis by inhibiting PI3k/AKT signaling (61). These three classical PTPs may synergistically participate in tumorigenesis by CNV-driven patterns in specific cancer. Therefore, we concluded that the dysregulation of classical PTPs by CNVs alterations might cause dysfunctions of pTyr and contribute to tumorigenesis in particular contexts.

We further presented a comprehensive study of the miRNAs-TFs-PTPs regulatory network. To our knowledge, this study shows the first report of the GRNs for classical PTPs across multiple tumor types based on experimental validation of TF and miRNA regulations. Thereby, we exhibited the topological properties of pan-cancer FFLs and revealed that PTPs in FFLs were more likely to be the hubs and bottlenecks. Furthermore, the specificity of GRNs in each cancer was identified and well documented to have essential roles in tumorigenesis. All these findings supported that GRNs were not only intensely connected in terms of network topology but also cancer prognosis and treatment. It is worth noting that this study is primarily based on TCGA RNA-seq data. However, the regulation of PTP is complex. Apart from the CNV, miRNA, and TF-mediated regulation examined in this work, epigenetic and post-translational alterations also play a significant role in PTPs in cancer. Given that our results are based on bioinformatic analysis, the function of the classical PTPs should be further confirmed and shown.

Finally, we explored the interactions of the classical PTPs with FDA-approved drugs and constructed the PTPscore to evaluate the impact of PTP activity on cancer progression. A surprising result is that 116 out of 118 CAGs are biased in at least one molecular signature layer across different cancer types. These CAGs are the targets of cancer treatments authorized by the FDA, such as immunotherapy, chemotherapy, hormone therapy, and targeted therapy. Our extensive analysis reveals that many CAGs are biased toward samples with a high PTPscore and supports that phosphatase-targeted treatment is promising cancer therapy, most likely as a component of combination therapy targeting CAGs. However, several phosphatase-targeted treatment clinical studies have had poor outcomes (7, 62–64), which is likely due to our imperfect knowledge of how molecular markers are impacted by the pTyr microenvironment and our lack of sensible combination therapy. Most therapeutic studies address the drug-resistant effects of pTyr, but pTyr may potentially increase treatment sensitivity in certain patients. These individuals may not experience therapeutic benefits from phosphatase-targeted therapy and/or combination therapies. Consequently, our systematic categorization of classical PTPs and identification of pTyr-biased signatures have significant therapeutic consequences; this research may aid in determining the clinical value of phosphatase-targeted treatment. We have exhibited the prevailing genetical and expression dysregulation of classical PTPs in human cancer. Classical PTPs are significantly associated with the activation and suppression of cancer-associated pathways and implicated with clinical prognosis. Taken together, the systematic panorama for the molecular hallmarks and clinical implications of classical PTPs provide a solid foundation for understanding the dysregulation of pTyr. It will further provide insights into the development of therapeutic strategies.

## Data availability statement

The original contributions presented in the study are included in the article/**Supplementary Material**. Further inquiries can be directed to the corresponding authors.

## Ethics statement

The studies involving human participants were reviewed and approved by the ethics committee of Bengbu medical college. The patients/participants provided their written informed consent to participate in this study. The animal study was reviewed and approved by the Animal Care and Use Committee of Northeastern University.

## Author contributions

BW and YY conceived and designed this work. TW, XB, XZ, and BB integrated and analyzed the bioinformatic data. TW, NZ, TL, JZ, and YZ performed the experiments and analyzed the data. XZ and GW collected the clinical specimens. TW and BW wrote and edited the manuscript. BW and YY reviewed the manuscript. All authors read and approved the final manuscript.

## Funding

This research was supported by the Liaoning Revitalization Talents Program (XLYC1902063), Key Research and Development Plan of Liaoning Province (2020JH2/10300080), National Natural Science Foundation of China (32100992, 31670770, 2016YFC1302402, 31370784), and the Fundamental Research Funds for the Central Universities of China (N2120001, N2120005).

## Conflict of interest

The authors declare that the research was conducted in the absence of any commercial or financial relationships that could be construed as a potential conflict of interest.

## Publisher’s note

All claims expressed in this article are solely those of the authors and do not necessarily represent those of their affiliated organizations, or those of the publisher, the editors and the reviewers. Any product that may be evaluated in this article, or claim that may be made by its manufacturer, is not guaranteed or endorsed by the publisher.
