# Supplementary figures and images for: Pan-cancer analyses of classical protein tyrosine phosphatases and phosphatase-targeted therapy in cancer
Source: Front Immunol. 2022 Oct 20;13:976996. doi: 10.3389/fimmu.2022.976996 (PMC9630847; doi:10.3389/fimmu.2022.976996)

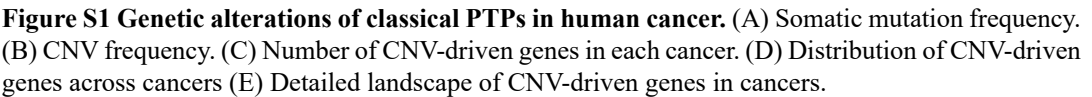

Supplement: Supplementary file 1 [file DataSheet_1.pdf]

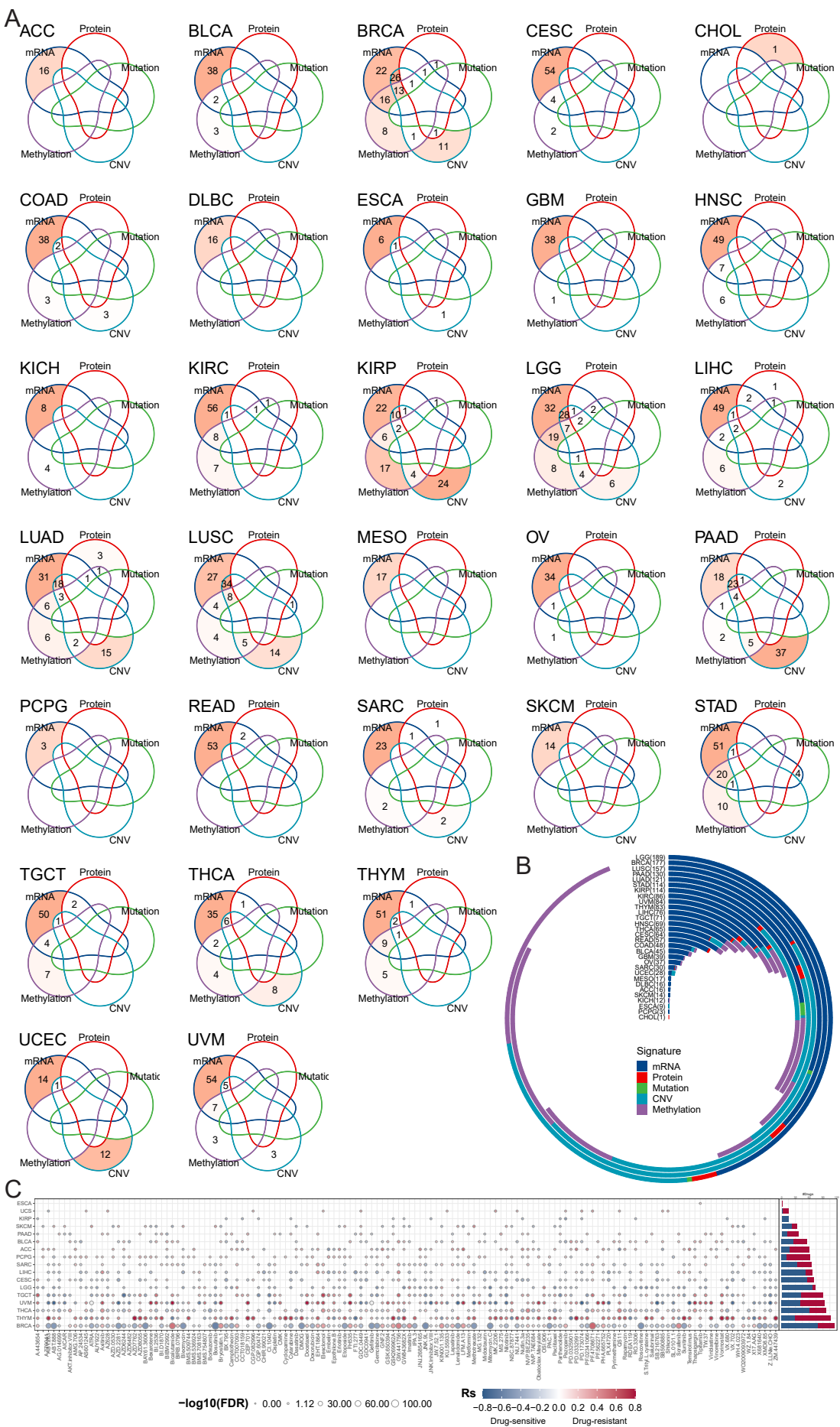

Supplement: Supplementary file 10 [file DataSheet_10.pdf]
