# Supplementary material for: Pan-cancer analyses of classical protein tyrosine phosphatases and phosphatase-targeted therapy in cancer
Source: Front Immunol. 2022 Oct 20;13:976996. doi: 10.3389/fimmu.2022.976996 (PMC9630847; doi:10.3389/fimmu.2022.976996)
Supplement: Supplementary file 3 [file DataSheet_3.pdf]

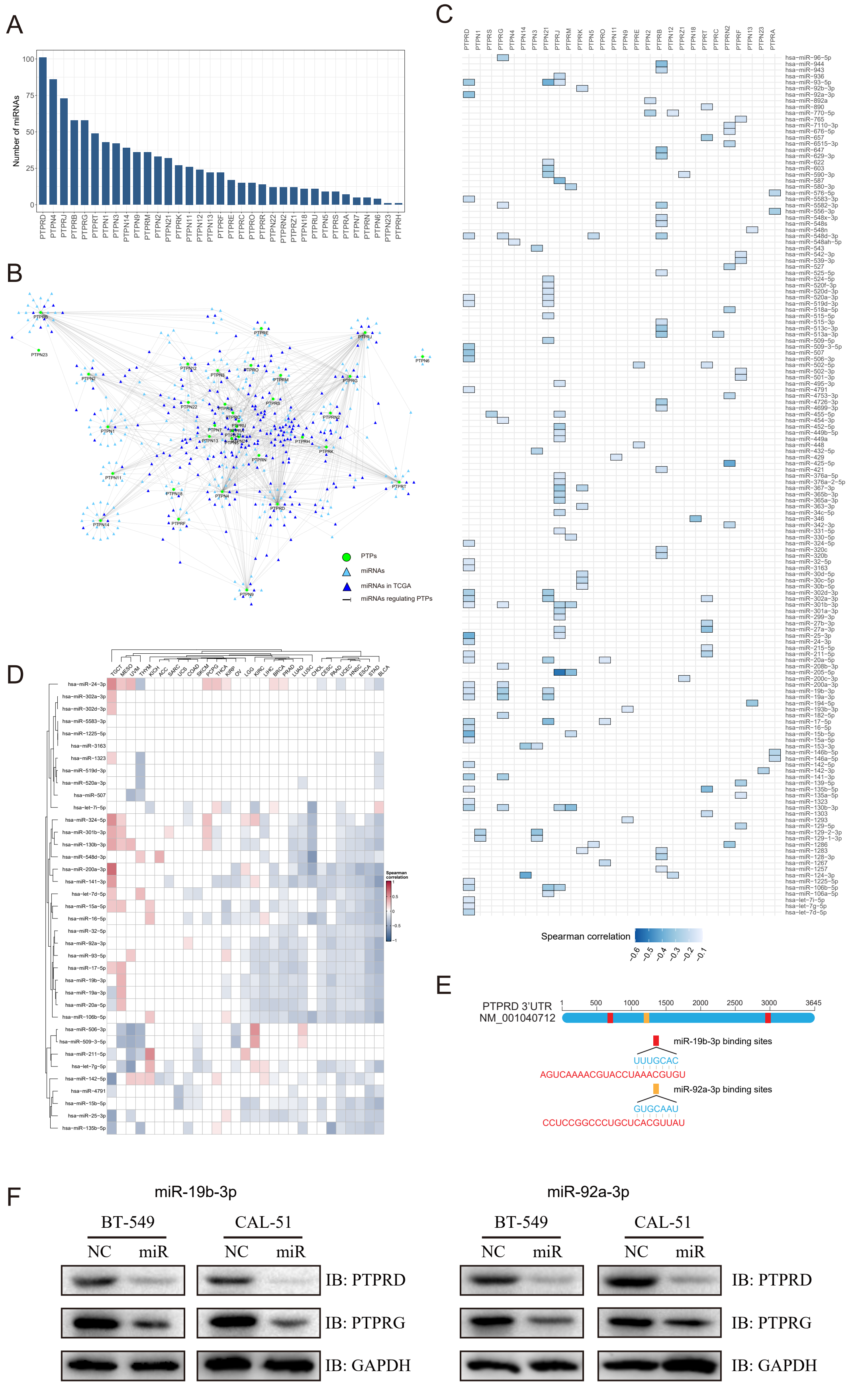

Figure S3. Landscape of miRNAs-PTPs pairs in human cancer. (A) Number of potential miRNAs for targeting their PTPs. (B) Interaction network of potential miRNAs and PTPs. (C) Regulation of classical PTPs by miRNAs in pan-cancer. (D) Regulation of PTPRD by miRNAs in different cancer. (E) PTPRD 3' UTR binding sites with miR-19b-3p and miR-92a-3p. (F) Relative protein expression of PTPs upon overexpression of target miRNAs.
