# Supplementary material for: Pan-cancer analyses of classical protein tyrosine phosphatases and phosphatase-targeted therapy in cancer
Source: Front Immunol. 2022 Oct 20;13:976996. doi: 10.3389/fimmu.2022.976996 (PMC9630847; doi:10.3389/fimmu.2022.976996)
Supplement: Supplementary file 4 [file DataSheet_4.pdf]

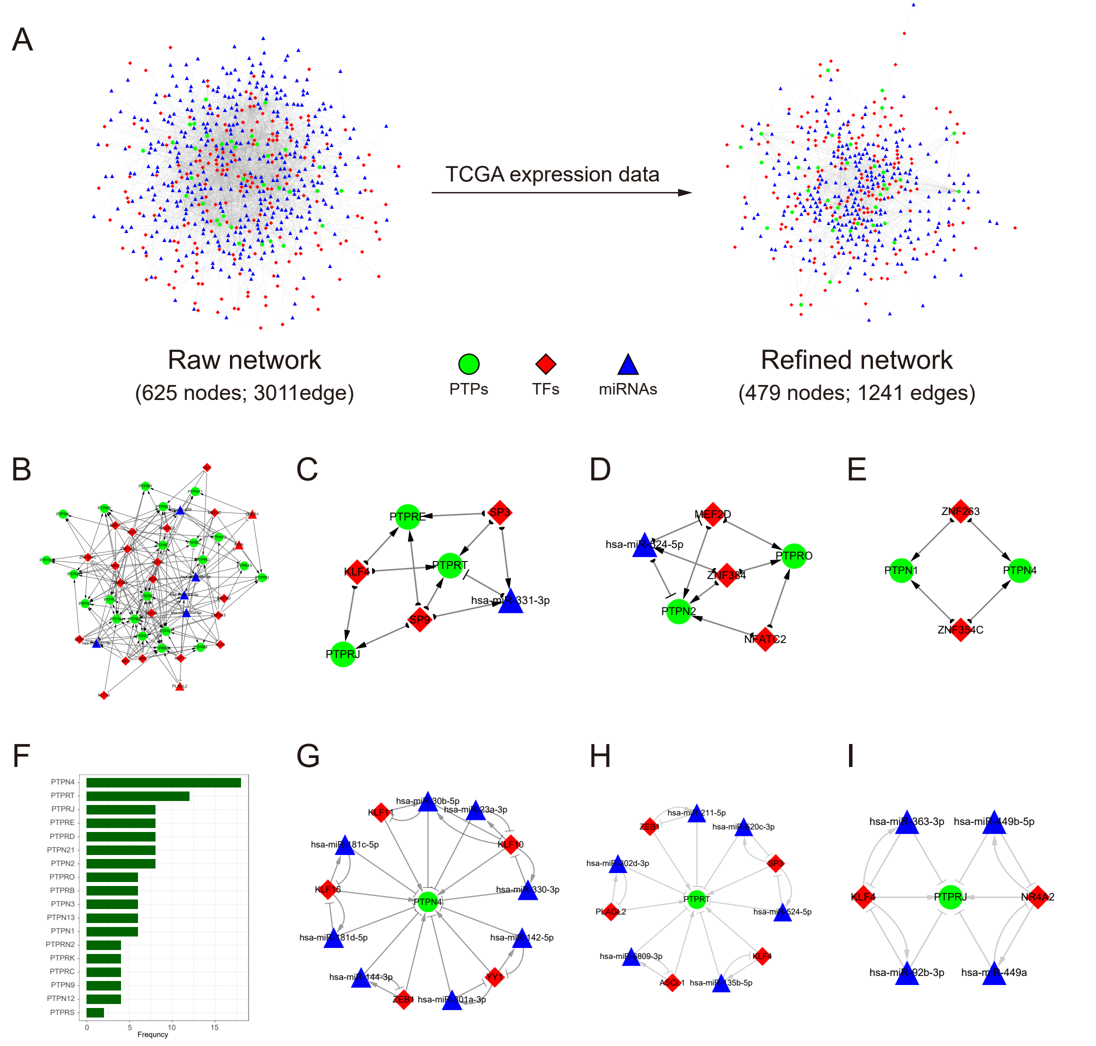

Figure S4. Characterization of FFLs interactive network. (A) Construction and refinement of the co-regulatory network. (B-E) Subnetwork and modules in network topology. (F) Frequency of PTPs in FFLs. (G-I) FFLs network involved in top frequency PTPs.
