# Supplementary material for: Pan-cancer analyses of classical protein tyrosine phosphatases and phosphatase-targeted therapy in cancer
Source: Front Immunol. 2022 Oct 20;13:976996. doi: 10.3389/fimmu.2022.976996 (PMC9630847; doi:10.3389/fimmu.2022.976996)
Supplement: Supplementary file 5 [file DataSheet_5.pdf]

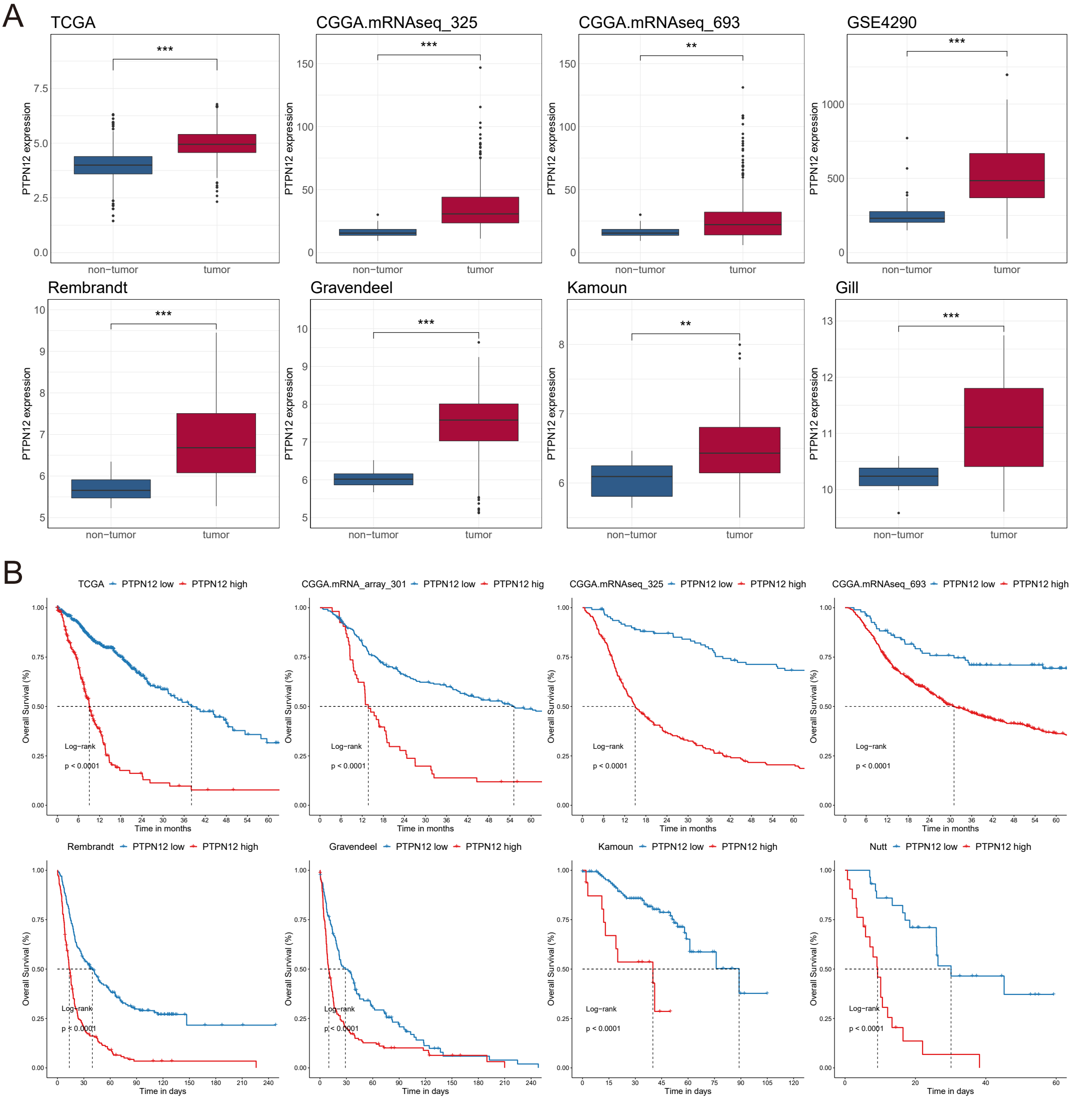

Figure S5. Independent validation of expression patterns and survival prognosis of PTPN12 in glioma. (A) PTPN12 expression in glioma from TCGA and three independent datasets. (B) Kaplan–Meier plot showing 5-year OS with the expression PTPN12 in glioma from TCGA and three independent datasets.
