# Supplementary material for: Pan-cancer analyses of classical protein tyrosine phosphatases and phosphatase-targeted therapy in cancer
Source: Front Immunol. 2022 Oct 20;13:976996. doi: 10.3389/fimmu.2022.976996 (PMC9630847; doi:10.3389/fimmu.2022.976996)
Supplement: Supplementary file 6 [file DataSheet_6.pdf]

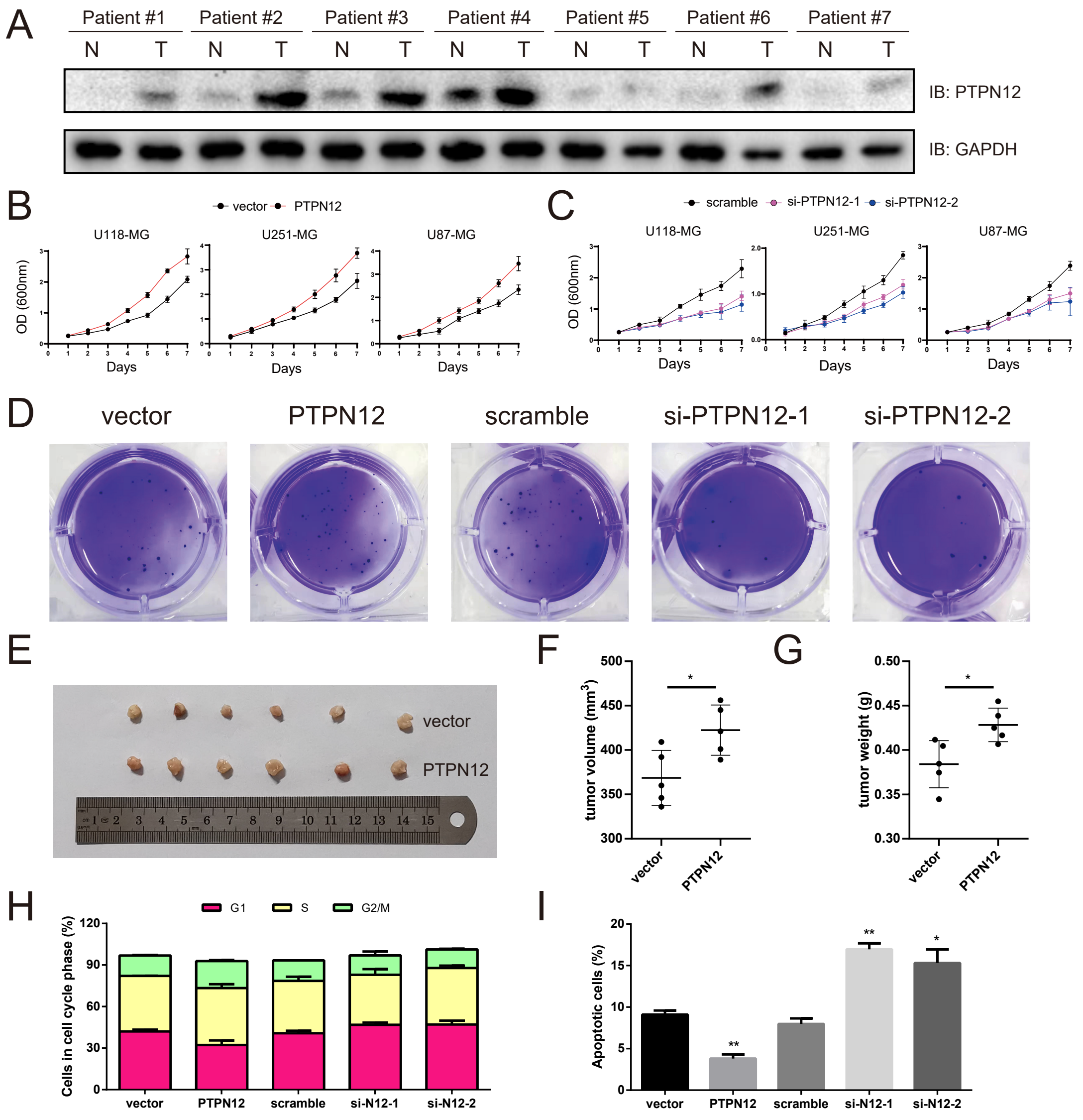

Figure S6. Functional validation of PTPN12 in glioma cells. (A) PTPN12 protein levels were detected in human glioma samples by western blot. N: normal; T: tumor. (B) Growth curves demonstrate the effect of ectopic expression of PTPN12 on glioma cell lines. (C) Growth curves demonstrate the effect of PTPN12 knockdown on glioma cell lines. (D) Colony formation in the cells following the indicating treatment. (E) Tumors were harvested and photographed from nude mice. (F-G) Final tumor volumes and weights were recorded and compared. (H) Effection of PTPN12 on cell cycle distribution. (I) Flow cytometric analysis of early and late apoptotic cells with annexin V and propidium iodide (PI).
