# Supplementary material for: Pan-cancer analyses of classical protein tyrosine phosphatases and phosphatase-targeted therapy in cancer
Source: Front Immunol. 2022 Oct 20;13:976996. doi: 10.3389/fimmu.2022.976996 (PMC9630847; doi:10.3389/fimmu.2022.976996)
Supplement: Supplementary file 7 [file DataSheet_7.pdf]

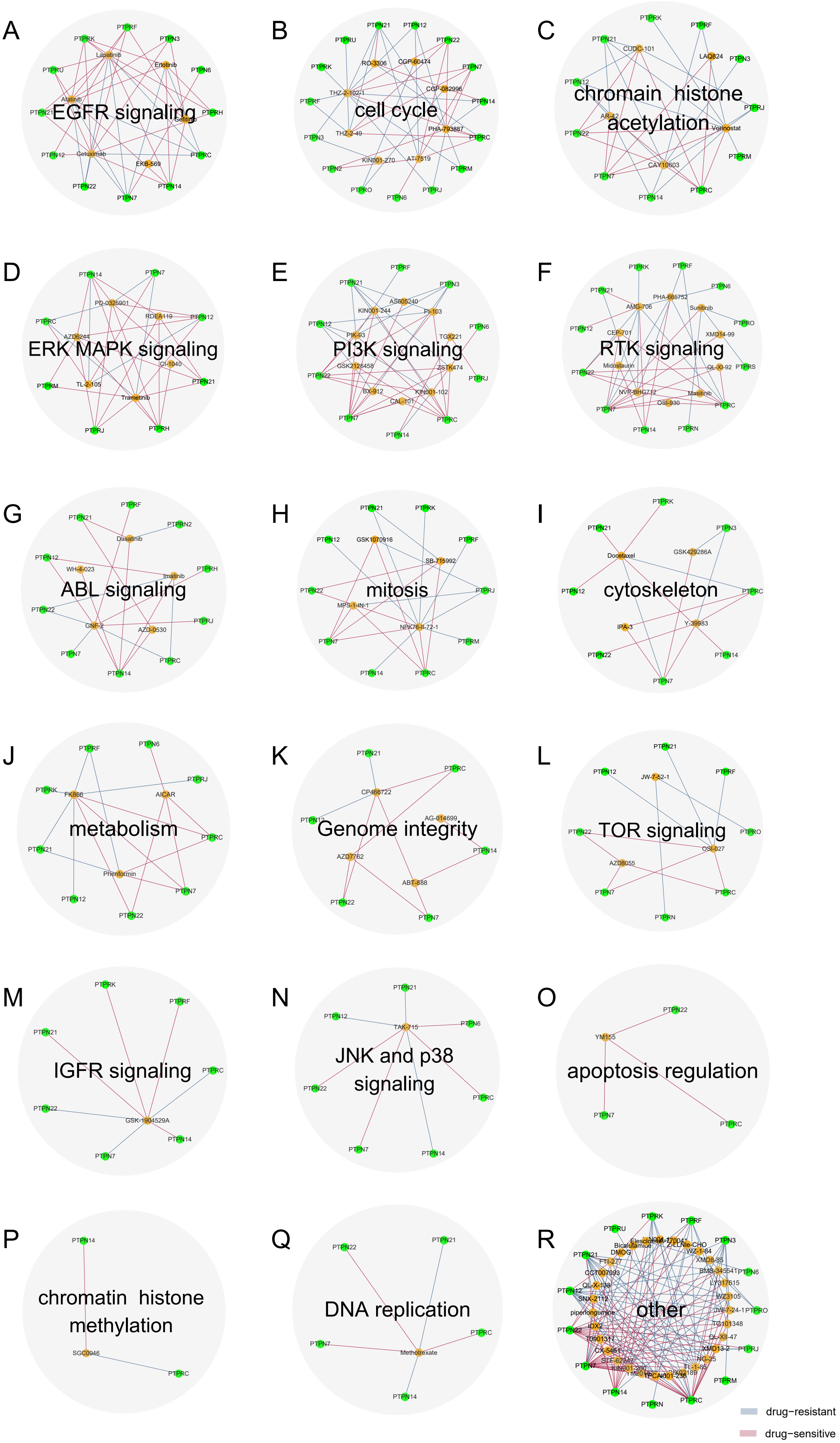

Figure S7. Classical PTPs (green point) correlate to drug-sensitive (blue) or drug-resistant (red) response to drug (orange point) involved in signaling pathways.
