# Supplementary material for: Pan-cancer analyses of classical protein tyrosine phosphatases and phosphatase-targeted therapy in cancer
Source: Front Immunol. 2022 Oct 20;13:976996. doi: 10.3389/fimmu.2022.976996 (PMC9630847; doi:10.3389/fimmu.2022.976996)
Supplement: Supplementary file 8 [file DataSheet_8.pdf]

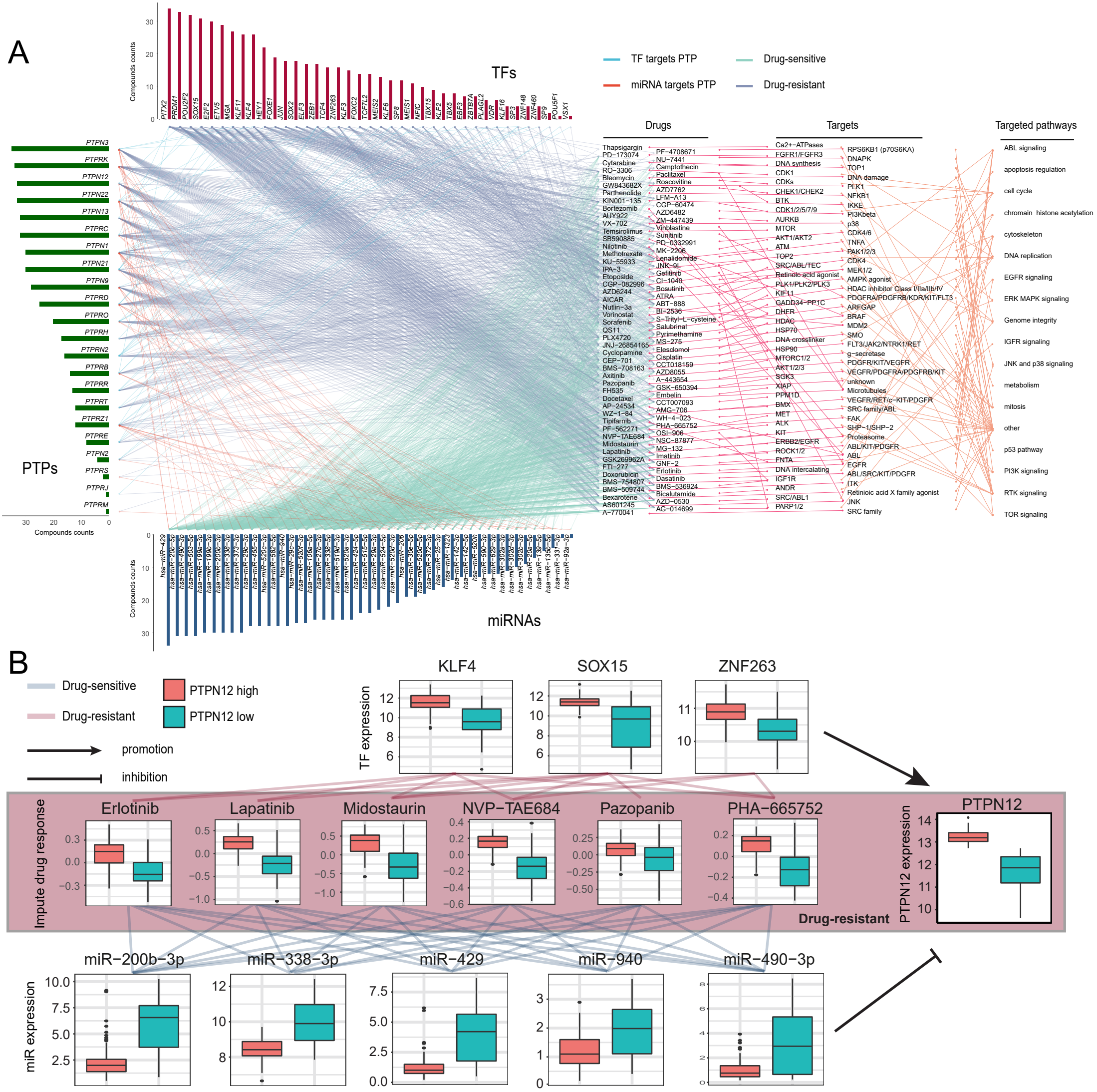

Figure S8. Significant correlation of classical PTPs to drug response in imputed data from TCGA. (A) Resistant interaction network of classical PTPs, PTP-targeted TFs, PTP-targeted miRNAs, and drug response in TGCT. (B) Representative drugs correlated to PTPN12, and corresponding TFs and miRNAs in TGCT.
