# Supplementary material for: Pan-cancer analyses of classical protein tyrosine phosphatases and phosphatase-targeted therapy in cancer
Source: Front Immunol. 2022 Oct 20;13:976996. doi: 10.3389/fimmu.2022.976996 (PMC9630847; doi:10.3389/fimmu.2022.976996)
Supplement: Supplementary file 2 [file DataSheet_2.pdf]

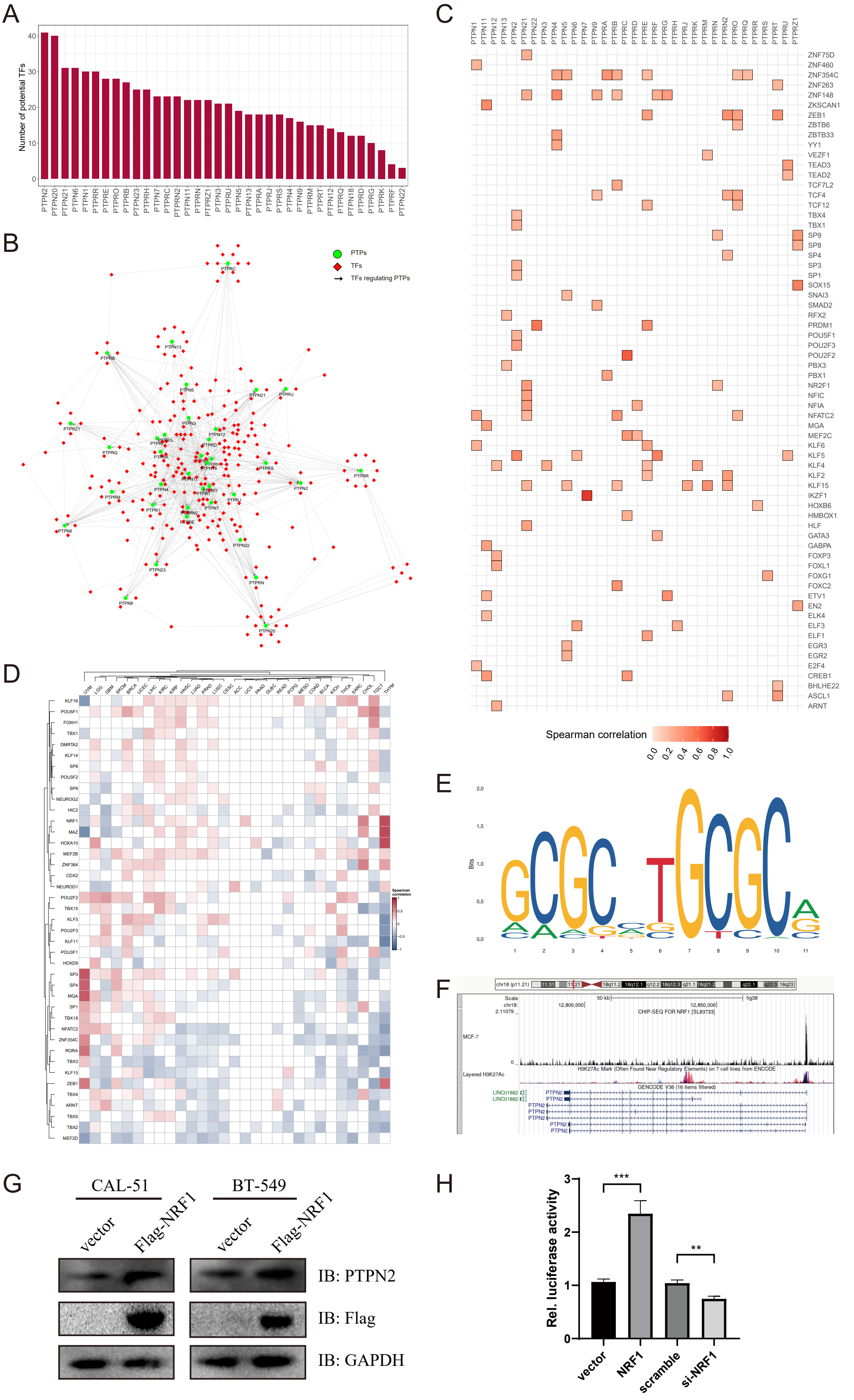

Figure S2. Landscape of TFs-PTPs pairs in human cancer. (A) Number of potential TFs for targeting their PTPs. (B) Interaction network of potential TFs and PTPs. (C) Regulation of classical PTPs by TFs in pan-cancer. (D) Regulation of PTPN2 by TFs in different cancer. (E) Motif sequence for NRF1. (F) Chip-seq data for potential regulation of PTPN2 by NRF1.(G) Relative protein expression of PTPN2 upon overexpression of NRF1. (H) Luciferase activity for target gene.
