# Supplementary material for: Pan-cancer analyses of classical protein tyrosine phosphatases and phosphatase-targeted therapy in cancer
Source: Front Immunol. 2022 Oct 20;13:976996. doi: 10.3389/fimmu.2022.976996 (PMC9630847; doi:10.3389/fimmu.2022.976996)
Supplement: Supplementary file 9 [file DataSheet_9.pdf]

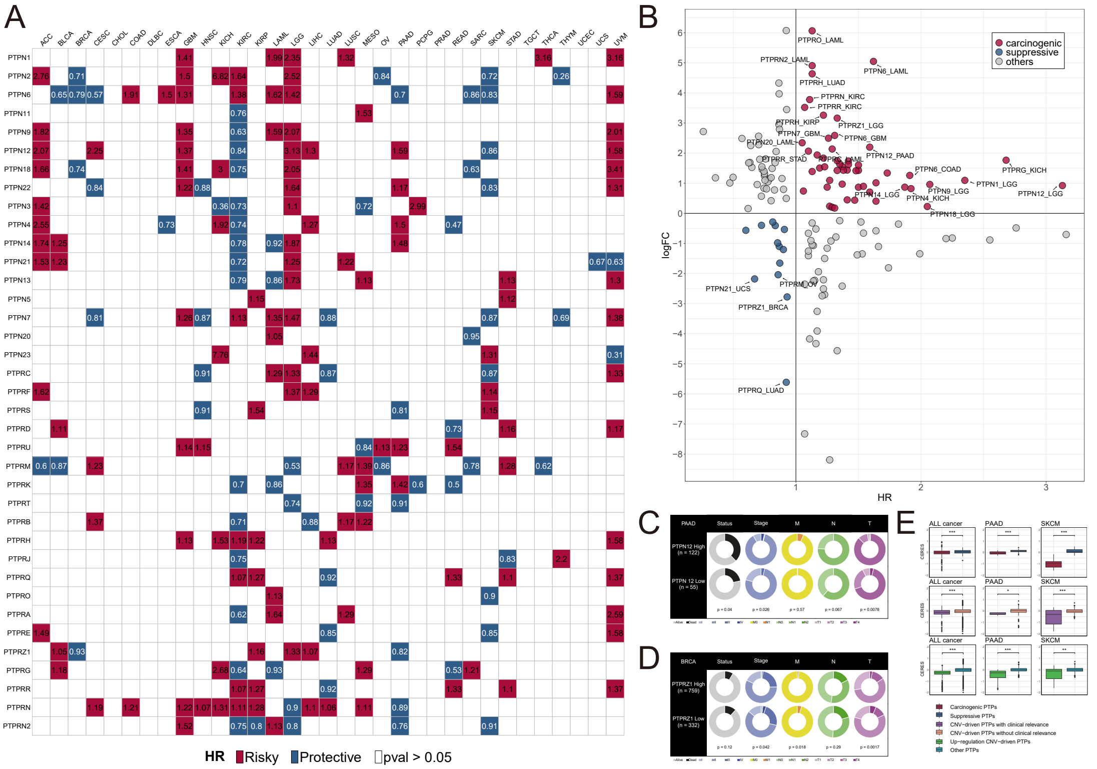

Figure S9. Clinical relevance of classical PTPs in human cancer. (A) Hazard ratio of classical PTPs across 33 types of cancer. (B) Potential cancerogenic properties of classical PTPs. (C-D) Clinicopathologic factors of target PTP in specific cancer. (E) CERES score for classical PTPs in cancer.
